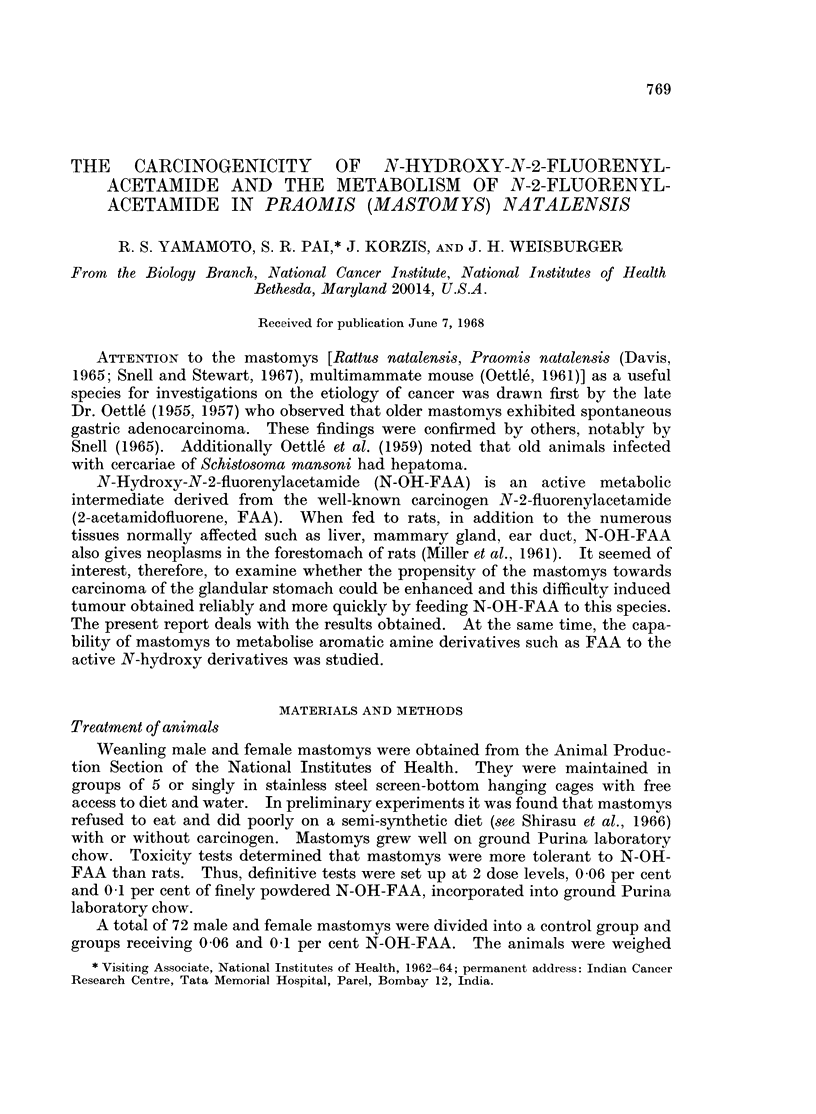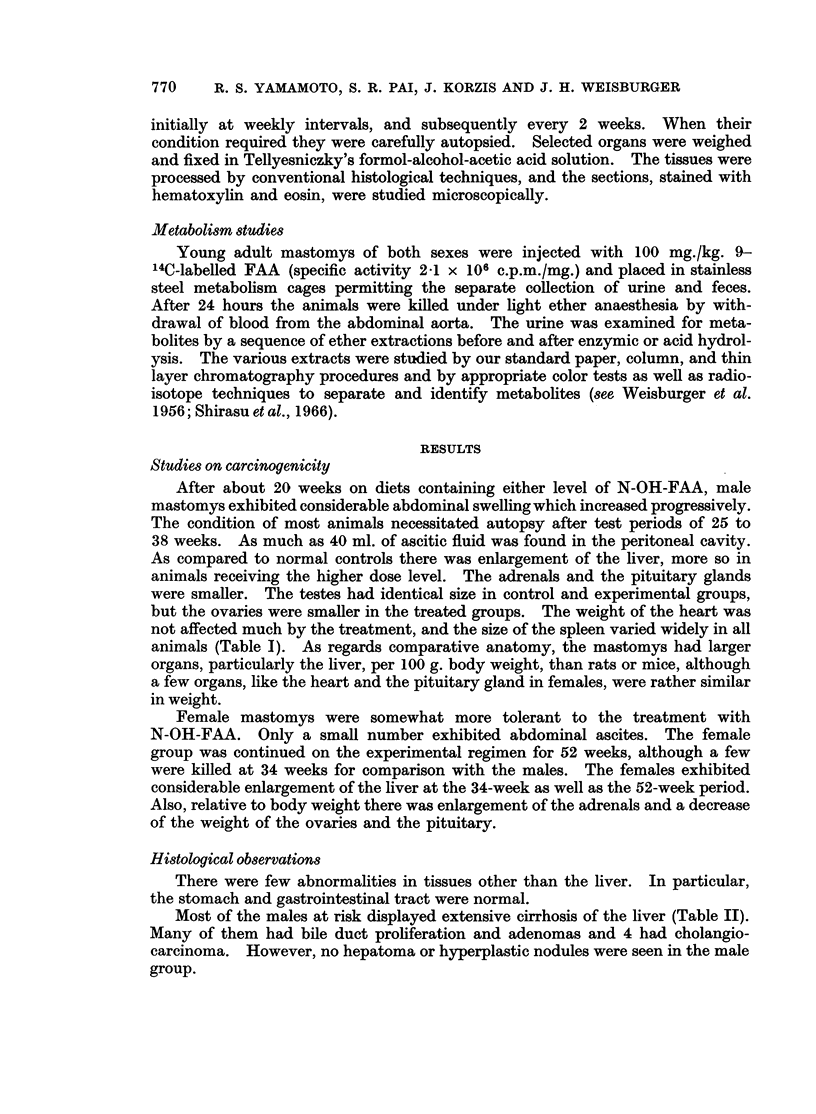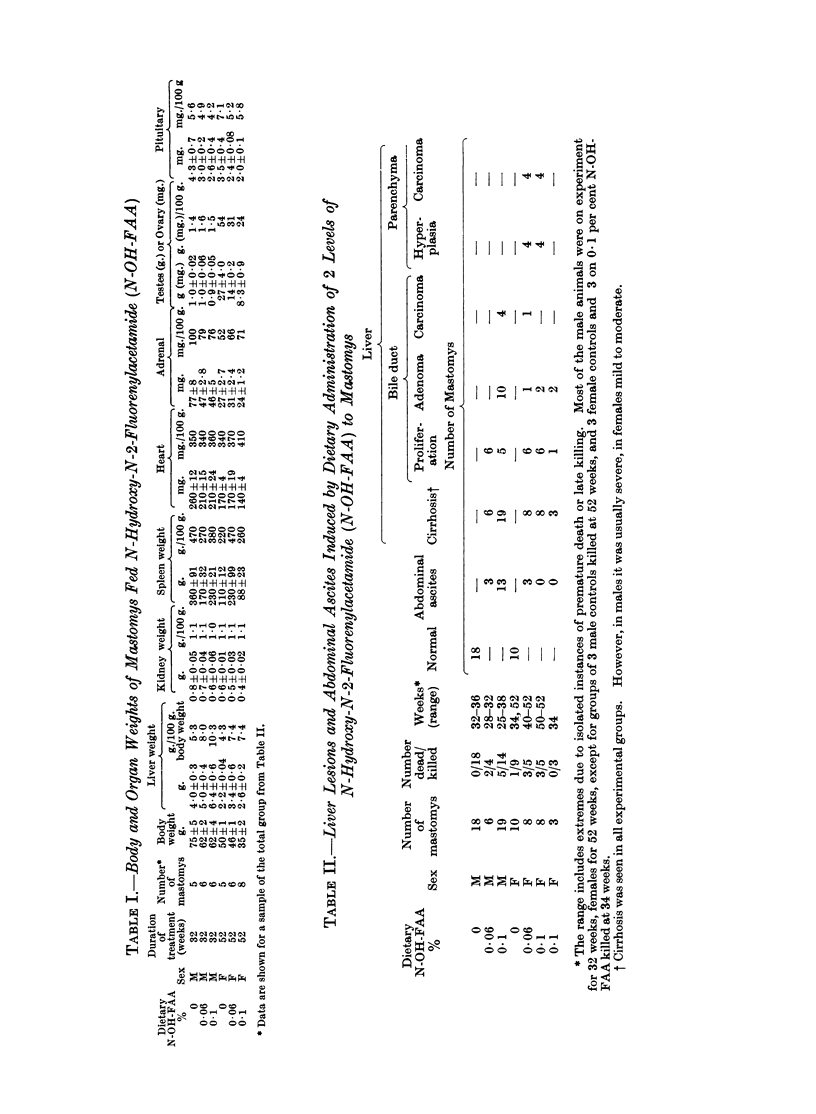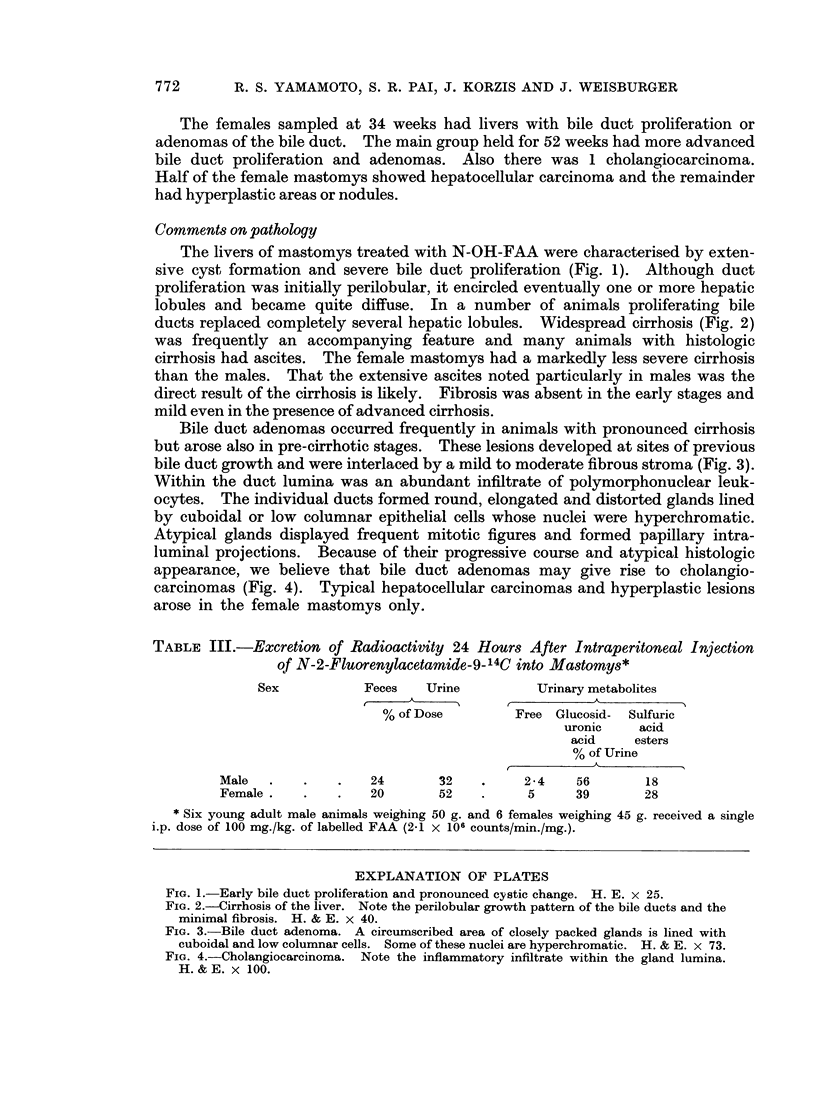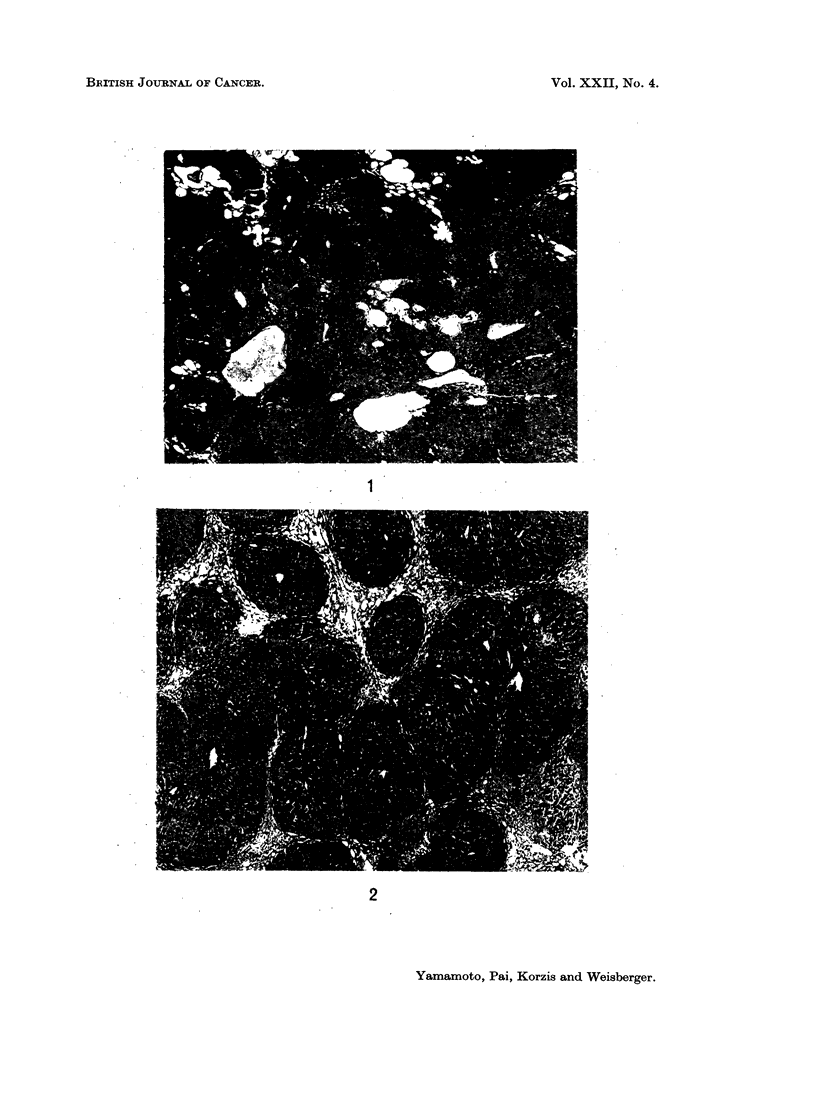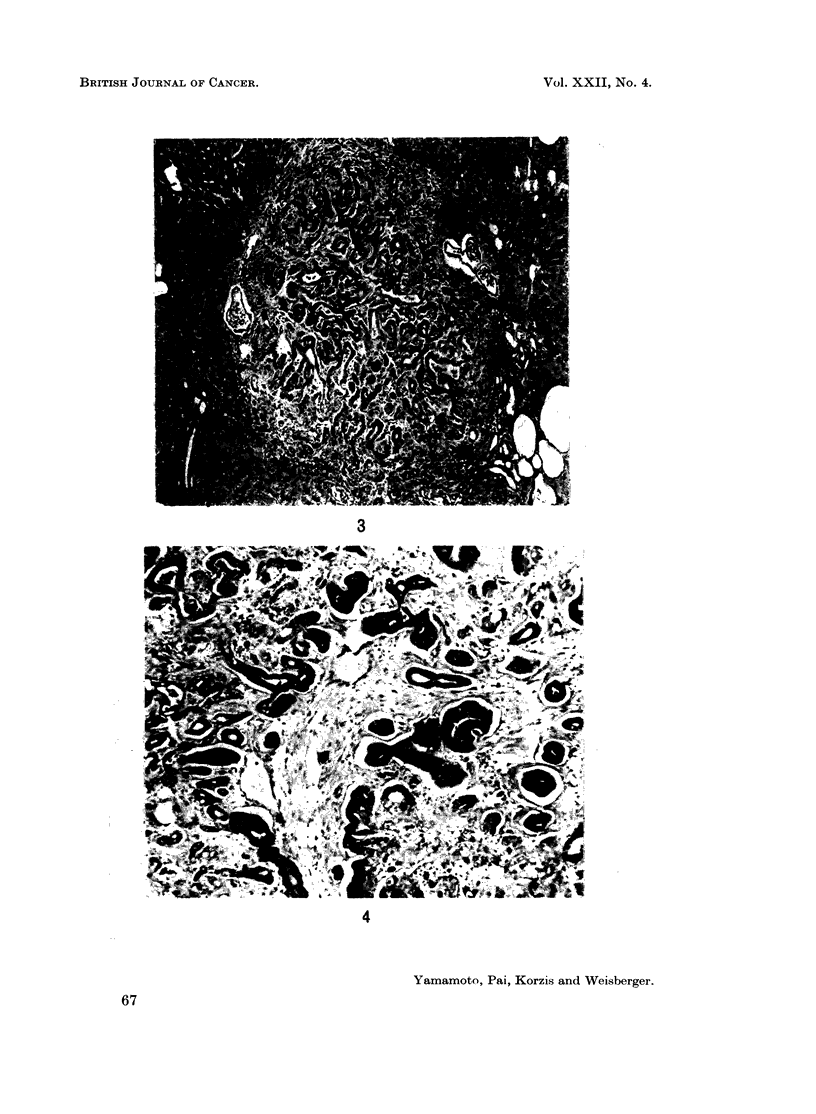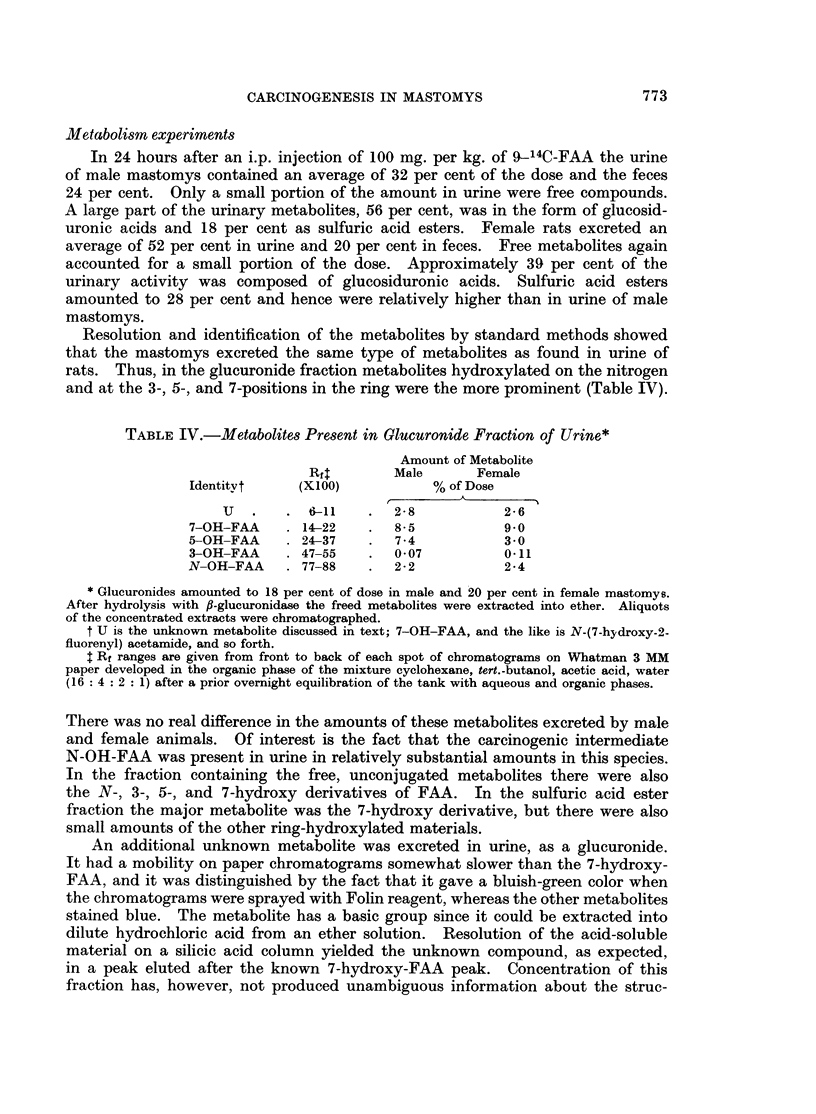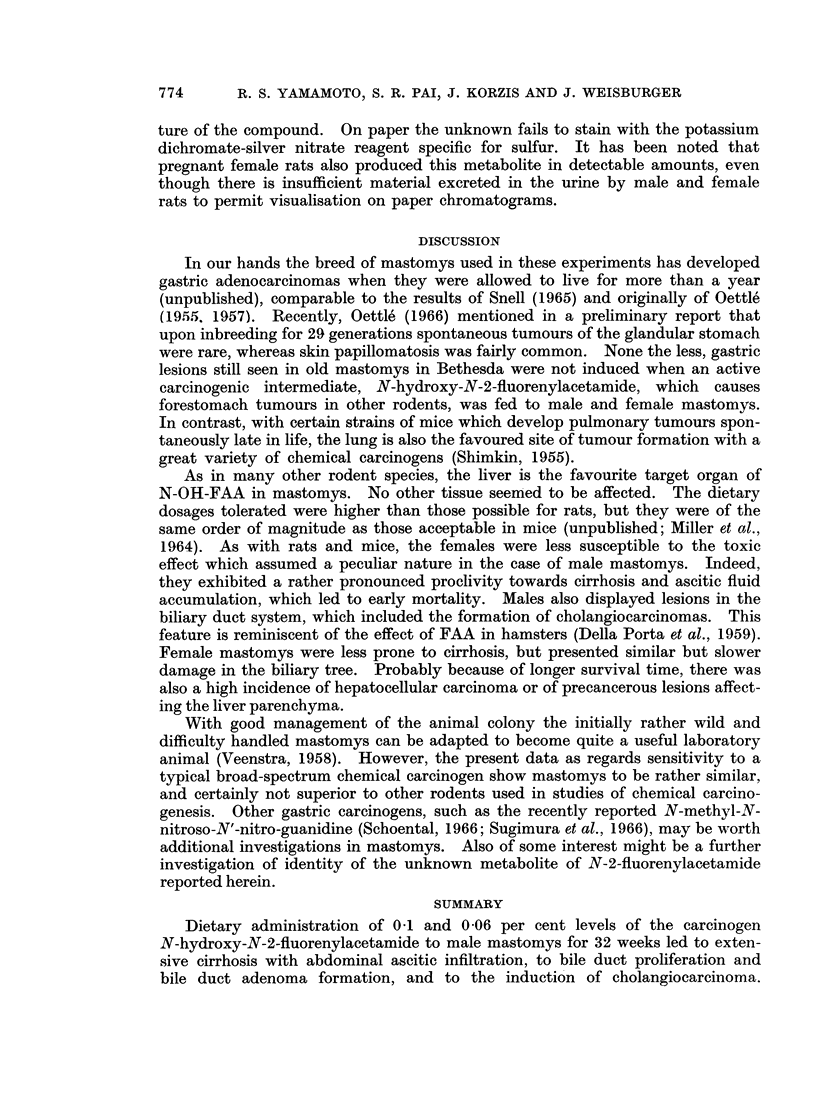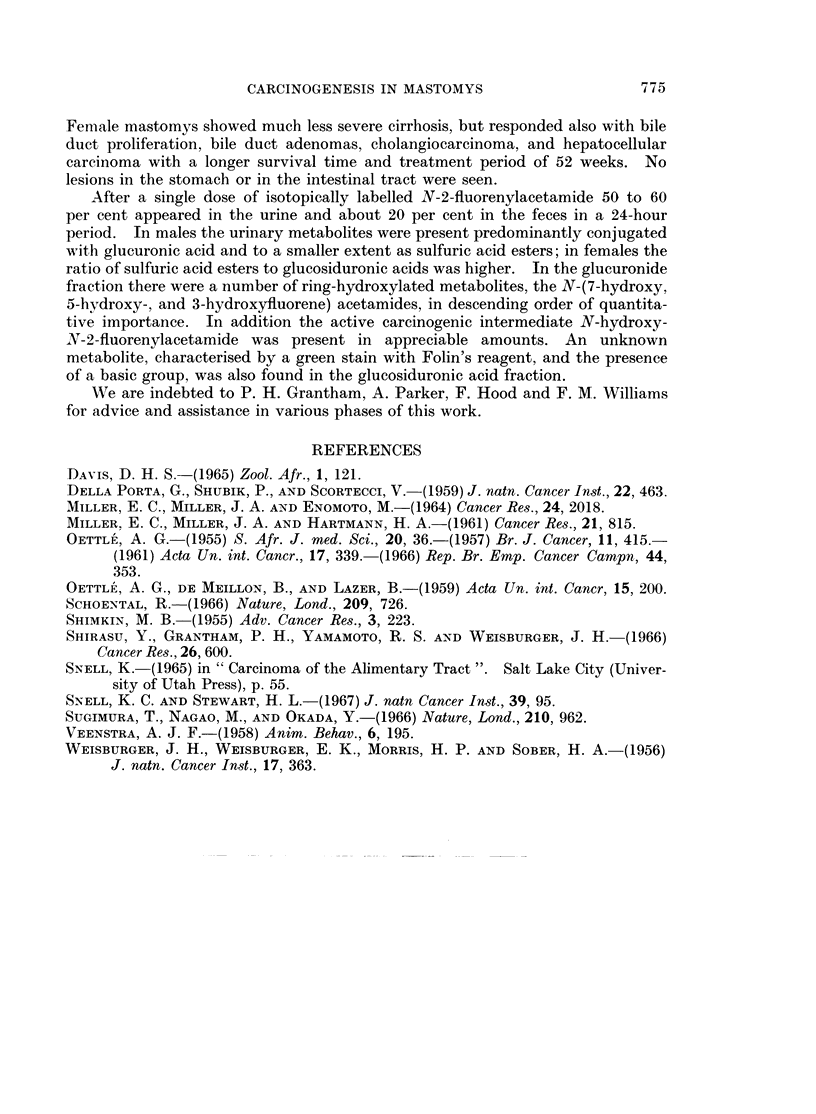# The carinogenicity of N-hydroxy-N-2-fluorenyl-acetamide and the matabolism of N-2-fluorenyl-acetamide in Praomis (mastomys) natalensis.

**DOI:** 10.1038/bjc.1968.91

**Published:** 1968-12

**Authors:** R. S. Yamamoto, S. R. Pai, J. Korzis, J. H. Weisburger

## Abstract

**Images:**


### THE CARCINOGENICITY OF *N*-HYDROXY-*N*-2-FLUORENYLACETAMIDE AND THE METABOLISM OF *N*-2-FLUORENYLACETAMIDE IN *PRAOMIS* (*MASTOMYS*) *NATALENSIS*

R. S. YAMAMOTO, S. R. PAI,* J. KORZIS, AND J. H. WEISBURGER

*From the Biology Branch, National Cancer Institute, National Institutes of Health Bethesda, Maryland* 20014, *U.S.A.*

Received for publication June 7, 1968

ATTENTION to the mastomys [*Rattus natalensis*, *Praomis natalensis* (Davis, 1965; Snell and Stewart, 1967), multimammate mouse (Oettlé, 1961)] as a useful species for investigations on the etiology of cancer was drawn first by the late Dr. Oettlé (1955, 1957) who observed that older mastomys exhibited spontaneous gastric adenocarcinoma. These findings were confirmed by others, notably by Snell (1965). Additionally Oettlé *et al.* (1959) noted that old animals infected with cercariae of *Schistosoma mansoni* had hepatoma.

*N*-Hydroxy-*N*-2-fluorenylacetamide (N-OH-FAA) is an active metabolic intermediate derived from the well-known carcinogen *N*-2-fluorenylacetamide (2-acetamidofluorene, FAA). When fed to rats, in addition to the numerous tissues normally affected such as liver, mammary gland, ear duct, N-OH-FAA also gives neoplasms in the forestomach of rats (Miller *et al.*, 1961). It seemed of interest, therefore, to examine whether the propensity of the mastomys towards carcinoma of the glandular stomach could be enhanced and this difficulty induced tumour obtained reliably and more quickly by feeding N-OH-FAA to this species. The present report deals with the results obtained. At the same time, the capability of mastomys to metabolise aromatic amine derivatives such as FAA to the active *N*-hydroxy derivatives was studied.

### MATERIALS AND METHODS

### *Treatment of animals*

Weanling male and female mastomys were obtained from the Animal Production Section of the National Institutes of Health. They were maintained in groups of 5 or singly in stainless steel screen-bottom hanging cages with free access to diet and water. In preliminary experiments it was found that mastomys refused to eat and did poorly on a semi-synthetic diet (*see* Shirasu *et al.*, 1966) with or without carcinogen. Mastomys grew well on ground Purina laboratory chow. Toxicity tests determined that mastomys were more tolerant to N-OH-FAA than rats. Thus, definitive tests were set up at 2 dose levels, 0·06 per cent and 0·1 per cent of finely powdered N-OH-FAA, incorporated into ground Purina laboratory chow.

A total of 72 male and female mastomys were divided into a control group and groups receiving 0·06 and 0·1 per cent N-OH-FAA. The animals were weighed

* Visiting Associate, National Institutes of Health, 1962–64; permanent address: Indian Cancer Research Centre, Tata Memorial Hospital, Parel, Bombay 12, India.

initially at weekly intervals, and subsequently every 2 weeks. When their condition required they were carefully autopsied. Selected organs were weighed and fixed in Tellyesniczky's formol-alcohol-acetic acid solution. The tissues were processed by conventional histological techniques, and the sections, stained with hematoxylin and eosin, were studied microscopically.

*Metabolism studies*

Young adult mastomys of both sexes were injected with 100 mg./kg. 9-$^{14}$C-labelled FAA (specific activity $2{\cdot}1 \times 10^6$ c.p.m./mg.) and placed in stainless steel metabolism cages permitting the separate collection of urine and feces. After 24 hours the animals were killed under light ether anaesthesia by withdrawal of blood from the abdominal aorta. The urine was examined for metabolites by a sequence of ether extractions before and after enzymic or acid hydrolysis. The various extracts were studied by our standard paper, column, and thin layer chromatography procedures and by appropriate color tests as well as radioisotope techniques to separate and identify metabolites (*see* Weisburger *et al.* 1956; Shirasu *et al.*, 1966).

### RESULTS

*Studies on carcinogenicity*

After about 20 weeks on diets containing either level of N-OH-FAA, male mastomys exhibited considerable abdominal swelling which increased progressively. The condition of most animals necessitated autopsy after test periods of 25 to 38 weeks. As much as 40 ml. of ascitic fluid was found in the peritoneal cavity. As compared to normal controls there was enlargement of the liver, more so in animals receiving the higher dose level. The adrenals and the pituitary glands were smaller. The testes had identical size in control and experimental groups, but the ovaries were smaller in the treated groups. The weight of the heart was not affected much by the treatment, and the size of the spleen varied widely in all animals (Table I). As regards comparative anatomy, the mastomys had larger organs, particularly the liver, per 100 g. body weight, than rats or mice, although a few organs, like the heart and the pituitary gland in females, were rather similar in weight.

Female mastomys were somewhat more tolerant to the treatment with N-OH-FAA. Only a small number exhibited abdominal ascites. The female group was continued on the experimental regimen for 52 weeks, although a few were killed at 34 weeks for comparison with the males. The females exhibited considerable enlargement of the liver at the 34-week as well as the 52-week period. Also, relative to body weight there was enlargement of the adrenals and a decrease of the weight of the ovaries and the pituitary.

*Histological observations*

There were few abnormalities in tissues other than the liver. In particular, the stomach and gastrointestinal tract were normal.

Most of the males at risk displayed extensive cirrhosis of the liver (Table II). Many of them had bile duct proliferation and adenomas and 4 had cholangiocarcinoma. However, no hepatoma or hyperplastic nodules were seen in the male group.

The females sampled at 34 weeks had livers with bile duct proliferation or adenomas of the bile duct. The main group held for 52 weeks had more advanced bile duct proliferation and adenomas. Also there was 1 cholangiocarcinoma. Half of the female mastomys showed hepatocellular carcinoma and the remainder had hyperplastic areas or nodules.

*Comments on pathology*

The livers of mastomys treated with N-OH-FAA were characterised by extensive cyst formation and severe bile duct proliferation (Fig. 1). Although duct proliferation was initially perilobular, it encircled eventually one or more hepatic lobules and became quite diffuse. In a number of animals proliferating bile ducts replaced completely several hepatic lobules. Widespread cirrhosis (Fig. 2) was frequently an accompanying feature and many animals with histologic cirrhosis had ascites. The female mastomys had a markedly less severe cirrhosis than the males. That the extensive ascites noted particularly in males was the direct result of the cirrhosis is likely. Fibrosis was absent in the early stages and mild even in the presence of advanced cirrhosis.

Bile duct adenomas occurred frequently in animals with pronounced cirrhosis but arose also in pre-cirrhotic stages. These lesions developed at sites of previous bile duct growth and were interlaced by a mild to moderate fibrous stroma (Fig. 3). Within the duct lumina was an abundant infiltrate of polymorphonuclear leukocytes. The individual ducts formed round, elongated and distorted glands lined by cuboidal or low columnar epithelial cells whose nuclei were hyperchromatic. Atypical glands displayed frequent mitotic figures and formed papillary intraluminal projections. Because of their progressive course and atypical histologic appearance, we believe that bile duct adenomas may give rise to cholangiocarcinomas (Fig. 4). Typical hepatocellular carcinomas and hyperplastic lesions arose in the female mastomys only.

TABLE III.—*Excretion of Radioactivity 24 Hours After Intraperitoneal Injection of N-2-Fluorenylacetamide-9-$^{14}$C into Mastomys**

| Sex | Feces (% of Dose) | Urine (% of Dose) | Urinary metabolites (% of Urine): Free | Glucosid-uronic acid | Sulfuric acid esters |
|---|---|---|---|---|---|
| Male | 24 | 32 | 2·4 | 56 | 18 |
| Female | 20 | 52 | 5 | 39 | 28 |

* Six young adult male animals weighing 50 g. and 6 females weighing 45 g. received a single i.p. dose of 100 mg./kg. of labelled FAA (2·1 × 10$^6$ counts/min./mg.).

### EXPLANATION OF PLATES

FIG. 1.—Early bile duct proliferation and pronounced cystic change. H. E. × 25.

FIG. 2.—Cirrhosis of the liver. Note the perilobular growth pattern of the bile ducts and the minimal fibrosis. H. & E. × 40.

FIG. 3.—Bile duct adenoma. A circumscribed area of closely packed glands is lined with cuboidal and low columnar cells. Some of these nuclei are hyperchromatic. H. & E. × 73.

FIG. 4.—Cholangiocarcinoma. Note the inflammatory infiltrate within the gland lumina. H. & E. × 100.

1

2

Yamamoto, Pai, Korzis and Weisberger.

3

4

Yamamoto, Pai, Korzis and Weisberger.

*Metabolism experiments*

In 24 hours after an i.p. injection of 100 mg. per kg. of 9–$^{14}C$-FAA the urine of male mastomys contained an average of 32 per cent of the dose and the feces 24 per cent. Only a small portion of the amount in urine were free compounds. A large part of the urinary metabolites, 56 per cent, was in the form of glucosiduronic acids and 18 per cent as sulfuric acid esters. Female rats excreted an average of 52 per cent in urine and 20 per cent in feces. Free metabolites again accounted for a small portion of the dose. Approximately 39 per cent of the urinary activity was composed of glucosiduronic acids. Sulfuric acid esters amounted to 28 per cent and hence were relatively higher than in urine of male mastomys.

Resolution and identification of the metabolites by standard methods showed that the mastomys excreted the same type of metabolites as found in urine of rats. Thus, in the glucuronide fraction metabolites hydroxylated on the nitrogen and at the 3-, 5-, and 7-positions in the ring were the more prominent (Table IV).

TABLE IV.—*Metabolites Present in Glucuronide Fraction of Urine**

| Identity† | $R_f$‡ (X100) | Amount of Metabolite Male | Female |
|---|---|---|---|
| | | % of Dose | |
| U | 6–11 | 2·8 | 2·6 |
| 7–OH–FAA | 14–22 | 8·5 | 9·0 |
| 5–OH–FAA | 24–37 | 7·4 | 3·0 |
| 3–OH–FAA | 47–55 | 0·07 | 0·11 |
| *N*–OH–FAA | 77–88 | 2·2 | 2·4 |

* Glucuronides amounted to 18 per cent of dose in male and 20 per cent in female mastomys. After hydrolysis with β-glucuronidase the freed metabolites were extracted into ether. Aliquots of the concentrated extracts were chromatographed.

† U is the unknown metabolite discussed in text; 7–OH–FAA, and the like is *N*-(7-hydroxy-2-fluorenyl) acetamide, and so forth.

‡ $R_f$ ranges are given from front to back of each spot of chromatograms on Whatman 3 MM paper developed in the organic phase of the mixture cyclohexane, *tert.*-butanol, acetic acid, water (16 : 4 : 2 : 1) after a prior overnight equilibration of the tank with aqueous and organic phases.

There was no real difference in the amounts of these metabolites excreted by male and female animals. Of interest is the fact that the carcinogenic intermediate N-OH-FAA was present in urine in relatively substantial amounts in this species. In the fraction containing the free, unconjugated metabolites there were also the *N*-, 3-, 5-, and 7-hydroxy derivatives of FAA. In the sulfuric acid ester fraction the major metabolite was the 7-hydroxy derivative, but there were also small amounts of the other ring-hydroxylated materials.

An additional unknown metabolite was excreted in urine, as a glucuronide. It had a mobility on paper chromatograms somewhat slower than the 7-hydroxy-FAA, and it was distinguished by the fact that it gave a bluish-green color when the chromatograms were sprayed with Folin reagent, whereas the other metabolites stained blue. The metabolite has a basic group since it could be extracted into dilute hydrochloric acid from an ether solution. Resolution of the acid-soluble material on a silicic acid column yielded the unknown compound, as expected, in a peak eluted after the known 7-hydroxy-FAA peak. Concentration of this fraction has, however, not produced unambiguous information about the structure of the compound. On paper the unknown fails to stain with the potassium dichromate-silver nitrate reagent specific for sulfur. It has been noted that pregnant female rats also produced this metabolite in detectable amounts, even though there is insufficient material excreted in the urine by male and female rats to permit visualisation on paper chromatograms.

### DISCUSSION

In our hands the breed of mastomys used in these experiments has developed gastric adenocarcinomas when they were allowed to live for more than a year (unpublished), comparable to the results of Snell (1965) and originally of Oettlé (1955. 1957). Recently, Oettlé (1966) mentioned in a preliminary report that upon inbreeding for 29 generations spontaneous tumours of the glandular stomach were rare, whereas skin papillomatosis was fairly common. None the less, gastric lesions still seen in old mastomys in Bethesda were not induced when an active carcinogenic intermediate, *N*-hydroxy-*N*-2-fluorenylacetamide, which causes forestomach tumours in other rodents, was fed to male and female mastomys. In contrast, with certain strains of mice which develop pulmonary tumours spontaneously late in life, the lung is also the favoured site of tumour formation with a great variety of chemical carcinogens (Shimkin, 1955).

As in many other rodent species, the liver is the favourite target organ of N-OH-FAA in mastomys. No other tissue seemed to be affected. The dietary dosages tolerated were higher than those possible for rats, but they were of the same order of magnitude as those acceptable in mice (unpublished; Miller *et al.*, 1964). As with rats and mice, the females were less susceptible to the toxic effect which assumed a peculiar nature in the case of male mastomys. Indeed, they exhibited a rather pronounced proclivity towards cirrhosis and ascitic fluid accumulation, which led to early mortality. Males also displayed lesions in the biliary duct system, which included the formation of cholangiocarcinomas. This feature is reminiscent of the effect of FAA in hamsters (Della Porta *et al.*, 1959). Female mastomys were less prone to cirrhosis, but presented similar but slower damage in the biliary tree. Probably because of longer survival time, there was also a high incidence of hepatocellular carcinoma or of precancerous lesions affecting the liver parenchyma.

With good management of the animal colony the initially rather wild and difficulty handled mastomys can be adapted to become quite a useful laboratory animal (Veenstra, 1958). However, the present data as regards sensitivity to a typical broad-spectrum chemical carcinogen show mastomys to be rather similar, and certainly not superior to other rodents used in studies of chemical carcinogenesis. Other gastric carcinogens, such as the recently reported *N*-methyl-*N*-nitroso-*N*′-nitro-guanidine (Schoental, 1966; Sugimura *et al.*, 1966), may be worth additional investigations in mastomys. Also of some interest might be a further investigation of identity of the unknown metabolite of *N*-2-fluorenylacetamide reported herein.

### SUMMARY

Dietary administration of 0·1 and 0·06 per cent levels of the carcinogen *N*-hydroxy-*N*-2-fluorenylacetamide to male mastomys for 32 weeks led to extensive cirrhosis with abdominal ascitic infiltration, to bile duct proliferation and bile duct adenoma formation, and to the induction of cholangiocarcinoma.

Female mastomys showed much less severe cirrhosis, but responded also with bile duct proliferation, bile duct adenomas, cholangiocarcinoma, and hepatocellular carcinoma with a longer survival time and treatment period of 52 weeks. No lesions in the stomach or in the intestinal tract were seen.

After a single dose of isotopically labelled *N*-2-fluorenylacetamide 50 to 60 per cent appeared in the urine and about 20 per cent in the feces in a 24-hour period. In males the urinary metabolites were present predominantly conjugated with glucuronic acid and to a smaller extent as sulfuric acid esters; in females the ratio of sulfuric acid esters to glucosiduronic acids was higher. In the glucuronide fraction there were a number of ring-hydroxylated metabolites, the *N*-(7-hydroxy, 5-hydroxy-, and 3-hydroxyfluorene) acetamides, in descending order of quantitative importance. In addition the active carcinogenic intermediate *N*-hydroxy-*N*-2-fluorenylacetamide was present in appreciable amounts. An unknown metabolite, characterised by a green stain with Folin's reagent, and the presence of a basic group, was also found in the glucosiduronic acid fraction.

We are indebted to P. H. Grantham, A. Parker, F. Hood and F. M. Williams for advice and assistance in various phases of this work.

### REFERENCES

DAVIS, D. H. S.—(1965) *Zool. Afr.*, **1**, 121.

DELLA PORTA, G., SHUBIK, P., AND SCORTECCI, V.—(1959) *J. natn. Cancer Inst.*, **22**, 463.

MILLER, E. C., MILLER, J. A. AND ENOMOTO, M.—(1964) *Cancer Res.*, **24**, 2018.

MILLER, E. C., MILLER, J. A. AND HARTMANN, H. A.—(1961) *Cancer Res.*, **21**, 815.

OETTLÉ, A. G.—(1955) *S. Afr. J. med. Sci.*, **20**, 36.—(1957) *Br. J. Cancer*, **11**, 415.—(1961) *Acta Un. int. Cancr.*, **17**, 339.—(1966) *Rep. Br. Emp. Cancer Campn*, **44**, 353.

OETTLÉ, A. G., DE MEILLON, B., AND LAZER, B.—(1959) *Acta Un. int. Cancr*, **15**, 200.

SCHOENTAL, R.—(1966) *Nature, Lond.*, **209**, 726.

SHIMKIN, M. B.—(1955) *Adv. Cancer Res.*, **3**, 223.

SHIRASU, Y., GRANTHAM, P. H., YAMAMOTO, R. S. AND WEISBURGER, J. H.—(1966) *Cancer Res.*, **26**, 600.

SNELL, K.—(1965) in " Carcinoma of the Alimentary Tract ". Salt Lake City (University of Utah Press), p. 55.

SNELL, K. C. AND STEWART, H. L.—(1967) *J. natn Cancer Inst.*, **39**, 95.

SUGIMURA, T., NAGAO, M., AND OKADA, Y.—(1966) *Nature, Lond.*, **210**, 962.

VEENSTRA, A. J. F.—(1958) *Anim. Behav.*, **6**, 195.

WEISBURGER, J. H., WEISBURGER, E. K., MORRIS, H. P. AND SOBER, H. A.—(1956) *J. natn. Cancer Inst.*, **17**, 363.